# Animal Plagues

**Published:** 1883-01

**Authors:** 


ANIMAL PLAGUES—THEIR HISTORY, NATURE AND PREVENTITION. By George Fleming, F.R.C.V.S., F.R.G.S., etc. Vol. II. (From A. D. 1800–1884:) London Bailliere, Tindall & Cox. 8vo., 539 pages. 1882.

One of the latest and most valuable contributions to veterinary science and literature is a work entitled "Animal Plagues," by George Fleming, F.R.C.V.S. This volume is a sequel to one published under the same title in 1871. In the first volume are to be found accounts of animal plagues, which occurred as early as B. C. 1490, and brings the history to the beginning of the present century.

The second volume continues the history onward from the year A, D. 1800.

The researches of this great veterinary author are very extensive, many of the accounts of the outbreaks of disease having never before appeared in English medical literature.

The coincident plagues of mankind are also embraced in the work, showing the intimate relationship which exists between the diseases of the lower animals and those that afflict the human race. Human practitioners as well as comparative physicians will welcome the appearance of this volume. The human physician fully realizes that in order to treat his patients from a scientific standpoint he is obliged to draw upon comparative medical science. The brute creation are subject to so many diseases that are similar in the human patient, and the human being contracting so many diseases, directly and indirectly, from animals of all species, the field of human medical science is widened to vast proportions. It is not denied that veterinarians have thrown much light upon many of the plagues of mankind.

The diseases of feral or untamed creatures and even fishes, are recorded and as much space as their importance calls for is allotted to them. At the end of the volume is appended a chronological synopsis of general diseases arranged according to species from B. C. 2048 to A. D. 1844.

Ignorance of the history of contagious diseases has been the cause of immense loss in our own country as well as in England. If more had been known about these plagues we would have had the necessary national as well as State legislation, and veterinarians would been supported in the work of their extermination.

